# Editorial: Mathematical Modelling and data analysis in infectious diseases

**DOI:** 10.3389/fpubh.2026.1975806

**Published:** 2026-09-22

**Authors:** Andrew Omame, Yanyu Xiao, Qing Han, Sarafa Adewale Iyaniwura

**Affiliations:** 1 Department of Mathematics and Statistics, York University, Toronto, ON, Canada; 2 Department of Mathematical Sciences, University of Cincinnati, Cincinnati, OH, United States; 3 Department of Mathematics, Augusta University, Augusta, GA, United States; 4 Vaccine and Infectious Disease Division, Fred Hutchinson Cancer Center, Seattle, WA, United States

**Keywords:** data-driven modeling, epidemic modeling, infectious diseases, mathematical modeling, statistical epidemiology

## Introduction

Infectious diseases spread through a complex mix of factors, including pathogens, hosts, behavior, mobility, environment, health systems, and interventions, all of which interact with each other. Mathematical models and data analysis are essential tools for understanding and scientifically simulating these interactions. These approaches enable researchers to draw robust conclusions from incomplete data, formalize competing hypotheses regarding pathogen transmissions and intervention effects, and integrate diverse sources of evidence to generate forecasts and support evidence-based decision-making. The coronavirus disease 2019 (COVID-19) pandemic accelerated the use of these tools on an unprecedented scale, with mathematical models informing decisions regarding lockdowns, vaccine allocations, and healthcare capacity-based planning in near real time. But the pandemic also made clear that good models need clear and evidence-based assumptions, must be tested against real data, should be checked against new data whenever possible, and must be used with an understanding of their uncertainty, not treated as perfect predictions (1–3). The challenge has only grown since: infectious-disease data are increasingly high-dimensional and multimodal, spanning genomic surveillance, mobility traces, wastewater signals, and electronic health records, while the methodological toolkit available to analyze them has expanded just as quickly, from classical compartmental models to machine learning and stochastic optimization.

The Research Topic *Mathematical Modeling and data analysis in infectious diseases* responds to this landscape by bringing together 22 contributions spanning public health, applied mathematics, infectious-disease epidemiology, and microbial sciences. The Research Topic covers a wide range of approaches, such as mechanistic modeling, statistical forecasting, machine learning, stochastic optimization, computational fluid dynamics, and burden analyses, applied across diseases including COVID-19, influenza, measles, mumps, dengue, and other pathogens of public health concern. Its unifying message is simple: no single method suffices for every question. Instead, the choice of method should follow from the decision at hand, the process generating the data, the observations actually available, and the consequences of incorrect inference or prediction.

## Robust inference and disease forecasting

Several contributions in this Research Topic address a central challenge in infectious-disease surveillance: extracting stable, decision-relevant predictions from data that evolve in real time. Rojas-Díaz et al. propose a robust calibration framework for delayed compartmental models, employing controlled parameter initialization and time-varying parameters to fit COVID-19 incidence, intensive-care burden, and effective reproduction numbers across Chilean regions. Their work underscores practical identifiability and numerical stability as prerequisites for translating mechanistic models into tools that can be used operationally in public health decision-making.

A complementary forecasting stream combines classical time-series methods with nonlinear learning. Xian et al. demonstrate that a SARIMA–*k*-nearest-neighbor (SARIMA-KNN) hybrid model improves prediction of acute hemorrhagic conjunctivitis in Chongqing, China, relative to its component models and Prophet-based comparators. Cao et al. integrated ARIMA and bidirectional long short-term memory networks through genetic-algorithm weighting, reporting improved COVID-19 forecasts for Japan with external validation in Germany. Alahmadi et al. applied seasonal-trend decomposition together with LightGBM to forecast post-pandemic influenza A in Saudi Arabia, illustrating the value of hybrid approaches when seasonal patterns are disrupted by structural change. Collectively, these studies point to an important direction for surveillance practice: hybrid models can disentangle recurrent linear or seasonal structure from irregular non-linear behavior, but their value depends on prospective evaluation and reporting forecasts alongside calibrated measures of uncertainty (3, 4).

The Research Topic also moves beyond single-location time series. One contribution introduces a spatiotemporal graph-learning framework that leverages geographic and origin-destination relationships to capture both contemporaneous neighborhood effects and lagged spatial spillovers in infectious-disease prediction (Liu et al.). Taken together with the forecasting studies above, this work underscores that local incidence is shaped not only by its own history but also by connections to other places, reporting processes, and shifting epidemic regimes. Beyond spatial and temporal structure, several contributions strengthen the statistical foundations needed for infectious-disease data that are non-normal, highly skewed, or operationally heterogeneous. Gharaibeh et al. introduced a two-parameter skewed Loai distribution for positive, right-skewed data and demonstrate its competitive fit to COVID-19 mortality data. At the healthcare-facility scale, Chen and Zhou compared SARIMA, ETS, Prophet, and NNETAR models for forecasting multidrug-resistant organism infection rates, showing that model choice should reflect both forecast accuracy and residual diagnostics rather than point-error measures alone.

## Disease transmission, interventions, and mobility

Mechanistic models offer a principled way to represent intervention timing, human behavior, and disease progression explicitly within the model structure. Babasola et al. formulated a time-delayed model for a potential human outbreak of highly pathogenic avian influenza, explicitly incorporating incubation, vaccine deployment, vaccine-to-protection delays, vaccine acceptance, residual susceptibility, and compliance with non-pharmaceutical interventions. Their simulations illustrate why early deployment and rapid protection are critical, and why non-pharmaceutical interventions remain especially important before vaccine-induced protection takes effect. Building on this theme, Ntaimo et al. developed an integrated chance-constrained stochastic-programming model for optimal COVID-19 vaccination and intervention strategies across multiple municipalities in Gauteng, South Africa. By incorporating uncertainty in contact patterns, household transmission, age-specific susceptibility and infectivity, and multiple vaccine-effectiveness endpoints, their approach makes risk tolerance an explicit part of allocation decisions. This work exemplifies a valuable bridge between epidemic modeling and operations research: the goal is not merely to simulate an outbreak but also to identify feasible actions under uncertainty.

Mobility is another recurring mechanism in this Research Topic. Drawing on cases from 294 Chinese cities, high-speed-rail records, migration data, and socioeconomic covariates, Zhang et al. found that rail connectivity was associated with early intercity dissemination of COVID-19 and that the Wuhan lockdown and transport suspensions disrupted this pathway. The importance of connectivity is echoed in a different setting: an age-structured, stochastic model of measles outbreaks in Northern British Columbia. Irvine et al. showed that inter-community mobility and school-based contact can increase outbreak probability and diminish the effectiveness of interventions, while the combined use of post-exposure prophylaxis and active case finding substantially reduces outbreak risk across scenarios.

At a finer spatial scale, Chong et al. combined epidemiological analyses of high-rise residences in Singapore with computational fluid-dynamics simulations, identifying elevated risk within vertical building stacks, and showed how wind direction and cross-ventilation can shape plausible aerosol transport pathways. Together, these contributions demonstrate that interventions must be conditioned on the structure of connectivity itself: rail networks, buildings, households, and social contacts each create distinct transmission pathways that call for correspondingly tailored control measures. Saha et al. extended this perspective across successive COVID-19 waves through a model-based hypothesis-testing framework that compares competing evolutionary explanations while estimating changes in transmission, virulence, vaccination, immunity, and contact patterns. Their analysis reinforces that epidemic trajectories emerge from the coupling of pathogen evolution and human response and that the dominant mechanism driving transmission may shift as population immunity and intervention conditions evolve.

## Environment, disease burden, and surveillance

A substantial group of papers demonstrates both the value and the interpretive challenges of linking disease surveillance with environmental, socioeconomic, and health-system data. He et al. assessed meteorological influences on influenza incidence in Wuxi, while Yao et al. examined environmental determinants of influenza-like illness across cities in Fujian Province between 2015 and 2023. Tao et al. characterized severe fever with thrombocytopenia syndrome and its meteorological associations in Jiangsu Province. Extending this line of work, Guo et al. applied generalized additive and distributed lag non-linear models to identify non-linear, interacting, and delayed meteorological associations with hemorrhagic fever with renal syndrome on China's Jiaodong Peninsula. Hao and Yu take a broader ecological view, using provincial panel models to examine bacillary dysentery in China and link incidence to healthcare capacity, economic development, and forest coverage. Together, these studies show that contextual covariates can strengthen local early warning and risk stratification when lagged, non-linear, and place-specific effects are properly accounted for; they also call for careful interpretation, since ecological and predictive associations do not, on their own, establish causation.

The Research Topic also broadens its scope from incidence to population-level burden. Luo et al. used Global Burden of Disease data to quantify trends, geographic inequalities, risk factors, and projected trajectories of childhood infectious encephalitis across Asia. Their results highlight persistent inequities, with lower socio-demographic development associated with higher disease burden, and underscore the continuing need to integrate surveillance, diagnostic capacity, perinatal care, environmental protection, and vaccination.

Gong et al. similarly used Global Burden of Disease data, inequality analysis, decomposition, age-period-cohort modeling, and forecasting to characterize the global burden of infectious diseases attributable to drug use. At a more local spatial scale, Hu et al. combined spatial autocorrelation analysis, spatial error models, and Geodetector methods to identify clustered gonorrhea incidence and its interacting sociodemographic, healthcare, and environmental correlates in China. Agrawal et al. synthesized evidence from 47 studies and 61 mumps outbreaks across 21 countries, estimating a pooled attack rate of 14.5% and a pooled complication rate of 10.3%, while documenting substantial heterogeneity by setting, age, and vaccination context. Their meta-analysis underscores the importance of standardized reporting and context-sensitive interpretation in an era of waning immunity and uneven vaccine access. More broadly, burden estimation and evidence synthesis connect model outputs to the equity questions that should guide infectious-disease policy.

###  Summary

This Research Topic brings together 22 contributions that showcase the breadth of mathematical and computational tools now used to understand and respond to infectious disease threats. Spanning mechanistic modeling, statistical forecasting, machine learning, stochastic optimization, computational fluid dynamics, and burden analysis, the Research Topic addresses diseases ranging from COVID-19 and influenza to measles, mumps, and dengue. Contributions cluster around three central themes: extracting robust, decision-relevant forecasts from noisy real-time surveillance data; modeling how interventions, mobility, and connectivity shape transmission dynamics; and linking disease burden to environmental, socioeconomic, and health-system determinants. Together, these papers reinforce a unifying message: no single method suffices for every question, and the right analytical approach must be matched to the decision at hand, the data-generating process, and the practical consequences of getting the inference wrong.
